# HypertenGene: extracting key hypertension genes from biomedical literature with position and automatically-generated template features

**DOI:** 10.1186/1471-2105-10-S15-S9

**Published:** 2009-12-03

**Authors:** Richard Tzong-Han Tsai, Po-Ting Lai, Hong-Jie Dai, Chi-Hsin Huang, Yue-Yang Bow, Yen-Ching Chang, Wen-Harn Pan, Wen-Lian Hsu

**Affiliations:** 1 Department of Computer Science and Engineering, Yuan Ze University, Chung Li, Taiwan, Republic of China; 2 Institute of Information Science, Academia Sinica, Nankang, Taipei, Taiwan, Republic of China; 3 Department of Computer Science, National Tsing-Hua University, HsinChu, Taiwan, Republic of China; 4 Institute of Biomedical Sciences, Academia Sinica, Nankang, Taipei, Taiwan, Republic of China

## Abstract

**Background:**

The genetic factors leading to hypertension have been extensively studied, and large numbers of research papers have been published on the subject. One of hypertension researchers' primary research tasks is to locate key hypertension-related genes in abstracts. However, gathering such information with existing tools is not easy: (1) Searching for articles often returns far too many hits to browse through. (2) The search results do not highlight the hypertension-related genes discovered in the abstract. (3) Even though some text mining services mark up gene names in the abstract, the key genes investigated in a paper are still not distinguished from other genes. To facilitate the information gathering process for hypertension researchers, one solution would be to extract the key hypertension-related genes in each abstract. Three major tasks are involved in the construction of this system: (1) gene and hypertension named entity recognition, (2) section categorization, and (3) gene-hypertension relation extraction.

**Results:**

We first compare the retrieval performance achieved by individually adding template features and position features to the baseline system. Then, the combination of both is examined. We found that using position features can almost double the original AUC score (0.8140vs.0.4936) of the baseline system. However, adding template features only results in marginal improvement (0.0197). Including both improves AUC to 0.8184, indicating that these two sets of features are complementary, and do not have overlapping effects. We then examine the performance in a different domain--diabetes, and the result shows a satisfactory AUC of 0.83.

**Conclusion:**

Our approach successfully exploits template features to recognize true hypertension-related gene mentions and position features to distinguish key genes from other related genes. Templates are automatically generated and checked by biologists to minimize labor costs. Our approach integrates the advantages of machine learning models and pattern matching. To the best of our knowledge, this the first systematic study of extracting hypertension-related genes and the first attempt to create a hypertension-gene relation corpus based on the GAD database. Furthermore, our paper proposes and tests novel features for extracting key hypertension genes, such as relative position, section, and template features, which could also be applied to key-gene extraction for other diseases.

## Background

The genetic factors leading to hypertension have been extensively studied, and large numbers of research papers have been published on the subject. Today, many hypertension researchers use PubMed to find and sort through papers of their interest, one of their primary research goals being to locate potentially hypertension-related genes. However, gathering such information with existing tools is not easy. For example, searching PubMed for hypertension-related articles often returns far too many hits to browse through. Second, the PubMed search results do not highlight the hypertension-related genes discovered in the abstract. Although there are text mining services that provide named entity recognition and mark up the gene names in an abstract, these systems do not distinguish the key genes that are the focus of research in the paper from other related genes that are merely mentioned.

To speed up and facilitate the information gathering process for hypertension researchers, one solution would be to list the key hypertension-related genes in each abstract alongside the abstract. In this paper, we construct a Hypertension key gene extraction system (HypertenGene) that adds the above functionality to PubMed via metasearch. Three key tasks need to be performed for support this system to work: (1) section categorization; (2) named entity (gene and hypertension names) recognition; (3) gene-hypertension relation extraction and ranking system.

### Related work

Section categorization is an important task for key gene extraction because the key hypertension-genes investigated in a paper tend to be mentioned in the results and conclusions sections. Other parts of the abstract, such as the introduction or background, may contain mentions of hypertension-related genes that nonetheless are not actually experimented upon in the paper.

Several approaches have been proposed in recent years. In 2003, Shimbo et al. [1] reported 91.9% accuracy using a support vector machine (SVM) model to classify sentences represented by bigrams and contextual information. Two years later, Yamamoto et al. [2] developed an SVM-based approach with various novel features, including subject-verb, verb tense, relative sentence location, and sentence score (i.e., the average TF-IDF (term frequency-inverse document frequency) score of words in a sentence) features. Their method achieved F-measures of 87.2% and 89.8% for the results and conclusion sections, respectively. Recently, Ruch et al. [3] used a Bayesian classifier with word and position features, which achieved an F-score of 85% in identifying the conclusion section of abstracts.

The above approaches explore several effective features for section categorization. However, they all share one potential weakness: they use a binary classifier to determine piece by piece which section each sentence belongs to without directly considering the labels of the sentence's neighbors in the whole abstract. This means that they sometimes misclassify non-contiguous sentences from the beginning, middle and end of an abstract into the same section. Though some types of post-processing can help catch these types of errors, the original model will still be flawed. The conditional random fields model labels the whole abstract at one time. That is, it assigns a tag sequence to a token sequence globally, not locally, which tackles the problems caused by token-by-token labeling. Several published papers have demonstrated that the CRF model generally achieves the best performance in the sequence labeling task [4]. Therefore, we have adopted the CRF model as the underlying machine learning model for the section categorization task.

Relation Extraction (RE) is the task of finding associations between entities within a given piece of text--sentence, paragraph, or entire document. The most popular approaches for relation extraction are rule-based [5], co-occurrence-based [6] and kernel-based [7]. In the biomedical field, most of the work in RE has been in identifying relations between proteins [6,8-12] identified relations between proteins and sub-cellular locations, and [13] extracted relations among cancer-related genes, drugs and cell-lines. Less work has been done on extracting relations among genes and diseases [14,15]; however, this area is now attracting increasing attention.

Some gene-disease relation extraction systems focus on the extraction of general gene-disease associations [13,16,17]. Other systems focus on discovering genes related to a specific disease only. For example, Tsujii et al.'s [18] maximum-entropy-based classifier extracted genes related to prostate cancer and gastric cancer. As a rule, general purpose systems are more portable, while disease-specific systems are able to exploit domain knowledge more thoroughly and thus achieve higher precision.

Granularity of relations is another aspect of relation extraction being explored. Some studies have attempted to extract and characterize the type of relation between entities [11,14]. Tsujii et al. [15] classifies gene-disease relations into two types: "etiology" and "clinical marker". Bundschus et al. [17] classified relations between genes and diseases into five types describing a wide variety of molecular conditions, ranging from genetic to transcriptional and phosphorylation events.

## Methods

In this section, we first introduce our system's two main components: named entity recognizers and section categorizer. Then, we illustrate the approach used in our main system: key hypertension gene extraction.

### Named Entity Recognizer

Our system extracts two NE types: gene and hypertension entities. Since gene named entities have many variations (e.g. Interleukin-2 can be written as "IL-2", "il-2", etc.), both internal and external contextual features should be exploited [19]. Machine learning-based approaches employ such features; therefore, we developed our gene NE recognizer based on our previous work--NERBio [20], a machine-learning-based NER system. In NERBio, the NER problem is formulated as a word-by-word sequence labeling task, where the assigned tags delimit the boundaries of any gene names. The underlying machine learning (ML) model used by NERBio is the conditional random fields model (CRF) [21], with a set of features selected by a sequential forward search algorithm. Unlike gene named entities, hypertension named entities do not have so many variations. We compiled a hypertension named entity dictionary by querying the MeSH term database with "hypertension". The database returned names of all diseases related to hypertension such as "high blood pressure", "gestational hypertension", and their synonyms. The maximum matching algorithm is employed to identify hypertension named entities in each given sentence. In addition, we also collect abbreviations of hypertension named entities (H') by matching the template H (H') with all abstracts in the gene association database (GAD), where H is any hypertension named entity in our dictionary.

### Section categorizer

**Figure 1:**
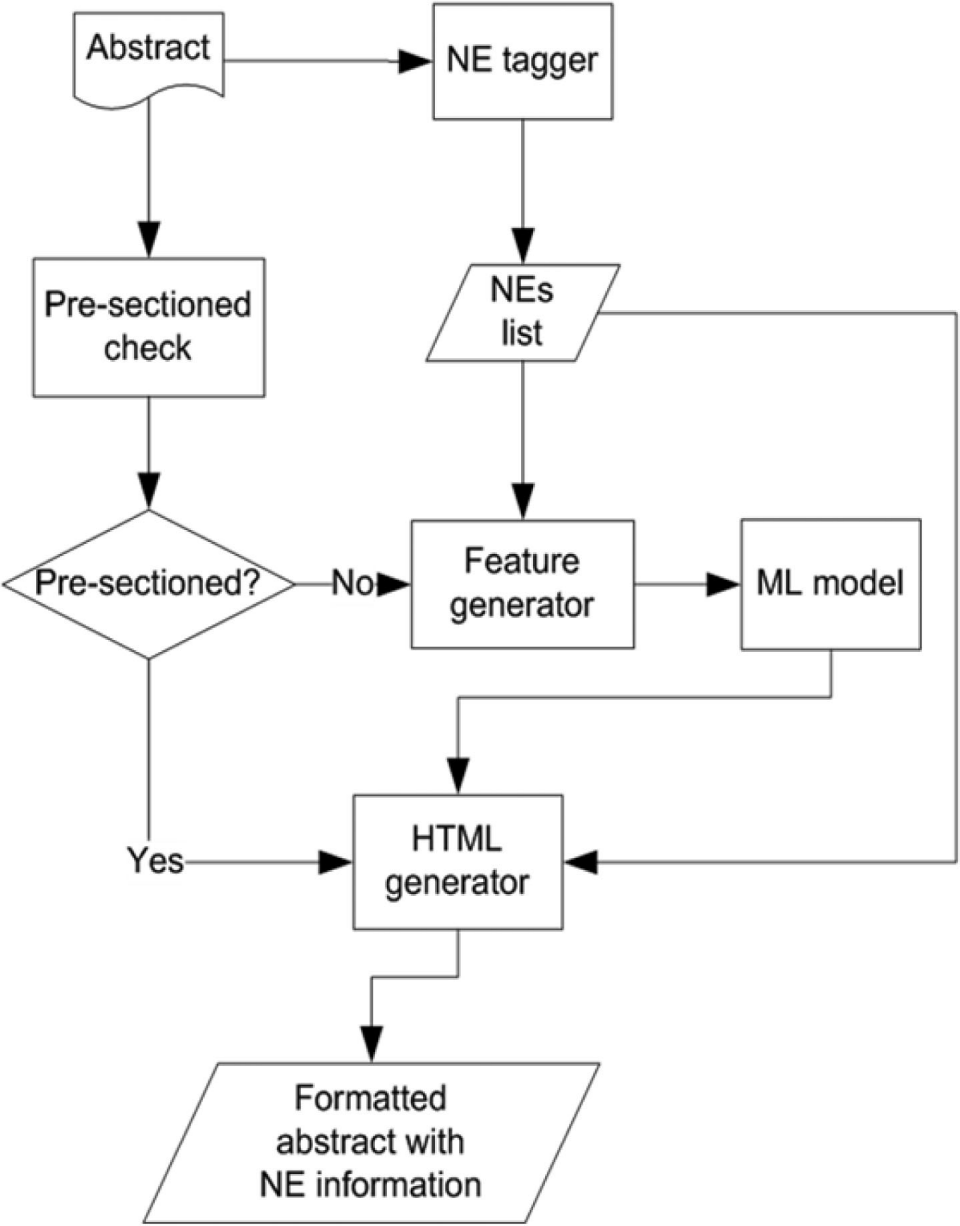
**Section categorizer flowchart**.

The function of this component is to divide a given abstract into section paragraphs. Figure 1 shows the flowchart of the section categorizer. For a given abstract, if the pre-sectioned check finds that the abstract contains obvious section tags, such as "Objective", and "Conclusion", the abstract is immediately divided into paragraphs. The pre-sectioned check uses a list of tag keywords collected by Hirohata et al. [22] to determine whether the abstract is pre-divided. The list keeps increasing every time our biologists or users submit new tags. If the check cannot find any obvious tags, a ML model is employed to section the given abstract.

For the section categorization problem, we regard each sentence in an abstract as a token. Each token is associated with a boundary tag, that is the beginning (*B*), inside (*I*) or outside (*O*) of a section, as well as a category tag, *c*, that indicates the category of the section. For example, in *B-c*, *I-c*, B- and, I- denote the first token and the subsequent token of a section in category *c*. Therefore, the problem can be formulated as the problem of assigning tags to each token. The underlying ML model is CRF. We describe three features of our model here. Other useful features for the section categorization problem can be found in our previous work [23].

The first is the 'tense feature'. Weissberg and Buker [24] suggested that an abstract has five important sections, "Background information", "Principal activity", "Methodlogy", "Results" and "Conclusion", which are often written in specific tenses. For example, the Results section is usually in past tense. We use ten part-of-speech (POS) patterns proposed in [23] to determine the tense of a given sentence. The second feature is the 'informative word feature', which determines the likelihood that certain words will appear in certain sections--for example "investigate" in the Objectives section, or "conclude" in the conclusion. The last feature is the 'title NE feature'. Since an abstract's title can be treated as a summary of the abstract, and NEs in the title often appear in the Results section, we can use co-occurrence frequency of NEs in the title and in a given sentence to identify the Results section.

Since there are no publicly available section categorization corpora, we constructed a corpus using the following procedure. Firstly, we compiled dozens of likely section headings from [22] into a list. Secondly, we searched PubMed using the keywords queries "hypertension" and "hypertension and (gene or DNA or RNA)" and compiled the approximately thirteen thousand results into a corpus after filtering out un-sectioned abstracts. Thirdly, we designed and implemented a program to remove section tags from the corpus abstracts. Finally, our in-lab biologists manually checked section boundaries in the collected abstracts.

To evaluate the performance of our section categorizer, we applied three-fold cross-validation using this corpus. Table 1 shows the evaluation results. As you can see, the categorizer achieves a satisfactory F-score of 98.82% with a precision of 98.77% and a recall of 98.87%.

**Table 1:** Section categorization performance

Section Type	P (%)	R (%)	F (%)
Objective	98.50	99.37	98.93
Method	98.54	97.74	98.14
Results	98.69	99.22	98.96
Conclusion	99.76	98.76	99.25

ALL	98.77	98.87	98.82

### Key hypertension related gene extraction

First, we briefly introduce the machine-learning model and baseline features used in our system. Then we explain our proposed features, which we discuss in the Results and Discussion sections, in more detail.

#### Formulation

For convenience, when referring to hypertension-gene pairs in the following explanations, we denote the hypertension named entity as H, the gene named entity as G, and the sentence containing the H-G pair as S. In this paper, our goal is to extract H-G relations from sentences in an abstract and rank them according to the degree of hypertension relatedness. We formulate the first subtask as a binary classification problem: determining if the target H-G pair is a key relation. All features of a target H-G pair are extracted from S. We then rank all extracted genes according their maximum H-G probability in an abstract as calculated by the classifier.

#### Maximum Entropy Model

The maximum entropy (ME) model is a flexible statistical framework that assigns an outcome to each instance on the basis of the history of that instance, which is made up of all the conditioning data that enable one to assign probabilities to the space of all outcomes. In H-G relation extraction, a history can be viewed as all the information related to the target pair that is derivable from the training corpus. ME computes the probability, *p*(*y*|*x*), for any *y *from the space of all possible outcomes, *Y*, and for every *x *from the space of all possible histories, *X*.

The computation of *p*(*y*|*x*) in an ME model depends on a set of binary features, which are useful for making predictions about the outcome. For instance, a pair that contains "associated with" between H and G is very likely to be hypertension related. Formally, we can represent this feature as follows:

Here, "Inter-HG_n-grams_associated(*x*)" is a binary function that returns a true value if the current sentence in the history, *x*, contains "associated" between H and G. KeyRelation corresponds to the class indicating that the H-G pair is a key relation in an abstract. Given a set of features and a training corpus, the ME estimation process produces a model in which every feature *f*_*i *_has a weight *α*_*i*_. We compute the conditional probability as follows:

The probability is calculated by multiplying the weights of the active features (i.e. those with *f*_*i *_(*x*,*y*) = 1); and *α*_*i *_is estimated by a procedure called Generalized Iterative Scaling (GIS) [25]. The ME estimation technique guarantees that, for every feature *f*_*i*_, the expected value of *α*_*i *_will equal the empirical expectation of *α*_*i *_in the training corpus. We use Zhang's MaxEnt toolkit and the L-BFGS [26] method of parameter estimation for our ME model.

#### Baseline features

Here, we describe the features used in our baseline model, which have all been employed extensively in previous studies [27-29].

##### Basic word features

There are two sets of word features used in our system, each with a different feature label.

1. Inter-HG n-grams

These features include all word unigrams and bigrams located between H and G. If none is present, the feature is given a "NULL" value.

2. Surrounding Words

These features include the two words before the first NE and the two after the second NE. If there are no words before or after both NEs, a "NULL" value is set. All words are treated as bag-of-words. That is, the order of these words is not considered.

##### Chunk features

Our system includes three sets of chunk features, each given a different feature label. We must first put all sentences through a shallow parser to capture phrase level information before chunk feature extraction.

1. Inter-HG chunk heads

Similar to surrounding word features, these chunk heads are treated as bag-of-words.

2. Surrounding chunk heads

These features include the two chunk heads to the left of the H-G pair and one chunk head to the right.

3. Inter-HG chunk types

##### Parse tree path features

We also parse each sentence with a full-sentence syntactic parser to generate its full parse tree. We can then use the syntactic path through the parse tree from the first NE to the second NE (not always H to G) as a feature.

**Figure 2:**
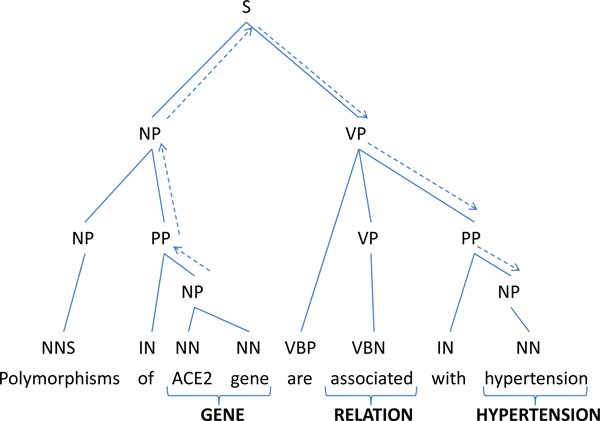
**Illustration of path NP↑PP↑NP↑S↓VP↓PP↓NP**.

For example, in Figure 2, the path from "ACE-2 gene" to "hypertension" is represented by the string NP↑PP↑NP↑S↓VP↓PP↓NP, where ↑ and ↓ represent upward and downward movement in the tree, respectively.

#### Proposed features

##### Template features

Although a few Inter-HG words and surrounding words tend to appear almost exclusively to true H-G relation pairs (e.g. "associate" for Inter-HG words), this information alone is not sufficient for argument-type classification for two reasons: (1) the collocation of surrounding words and Inter-HG words is a more precise indicator of a true H-G relation then using Inter-HG words; and (2) the window of surrounding words is limited to 2, making some important surrounding words are missed, but expanding the window size may introduce more noise words. Templates composed of unlimited surrounding words and Inter-HG words can be helpful for identifying the argument type of a constituent.

**Figure 3:**
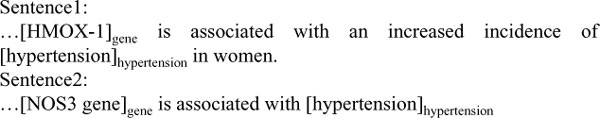
**Example of paired similar sentences containing H-G pairs**.

Our template generation (TG) algorithm, which extracts syntactic patterns for H-G pairs using Smith and Waterman's local alignment algorithm [30], starts by pairing all sentences containing true relations according to their similarity. Closely-matched pairs are then aligned word-by-word and a pattern satisfying the alignment result is created. Each slot in the template is given by the corresponding constraint information expressed in the form of a word (e.g. "associated"). If two aligned sentences have nothing in common for a given slot, the TG algorithm puts a wildcard in the position. Figure 3 shows a pair of aligned arguments from which the TG algorithm generated the template "<gene> * is associated with * <hypertension>". In the first position both sentences share a common NE type (gene); in the 2nd to 4th positions they have the same phrase, "is associated with"; and in the end position they share a common NE type, hypertension. The complete TG algorithm is described with pseudo code in Algorithm 1. The similarity function used to compare the similarity of two tokens in Smith and Waterman's algorithm is defined as:

where *x *and *y *are tokens in sentences *s*_*i *_and *s*_*j*_, respectively. The similarity of two sentences is calculated by the Smith and Waterman algorithm on the basis of this token-level similarity function.

**Algorithm 1 **Template generation

Input: A set of sentences *A *= {*s*_1_,...,*s*_*k*_},

Output: A set of templates *T *= {*t*_1_,...,*t*_*k*_}.

1: *T *= {};

2: **for **each sentence *s*_*i *_from *s*_1 _to *s*_*n*-1 _**do**

3:   **for **each sentence *s*_*j *_from *s*_*i *_to *s*_*n *_**do**

4:      **if **the similarity of *s*_*i *_and *s*_*j *_calculated by an alignment is above the threshold *τ*

5:      **then **generate a common template ***t ***for *s*_*i *_and *s*_*j*_;

7:      *T*←*T*∪***t***;

8:   **end**;

9: **end**;

10: return *T*;

Based on template matching, we develop two feature types.

1. Positive Match

If S matches any patterns and there is no negative word in it, this feature is enabled.

2. Negative Match

Unlike the Positive Match feature, if S matches any patterns but there is at least one negative word, such as "not", "fail", "unable", "inability", "neither", "nor", "failure", and etc. in it, this feature is enabled.

##### Position Features

1. Relative position features

Because the focused H-G pair is usually located at the end of the abstract, the relative position of a sentence in an abstract provides useful information. Therefore, we define the following equation to represent the relative position of a sentence:

Since ME can only handle discrete feature values, we set ten binary features corresponding to the relative position function's possible values ranging between 1 and 10.

2. Section features

These features indicate in which section S is located. S's section is calculated by the above mentioned section categorizer. There are four section features: Objective, Methods, Results, Conclusion.

## Results and discussion

### Datasets

Currently, there are no publicly available annotated corpora for training gene-hypertension relation extraction systems. To create one, we took sentences from the gene association database (GAD) [31], which is curated by the National Institute on Aging. The GAD data consist of PubMed IDs and the diseases and related genes mentioned in the articles. In comparison to PubMed, which contains a variety of articles on everything from clinical trials to literature surveys, the GAD focuses only on experimental reports of gene-disease relations.

Our data set consists of 939 sentences retrieved from 195 randomly selected abstracts of hypertension-related papers listed in the GAD. Our goal is to extract etiological relations among genes and diseases in those sentences. Since the disease and gene names associated with each article are not annotated in the retrieved abstracts, we must first employ our hypertension named-entity recognizer and gene named-entity recognizer introduced in the Methods section to automatically annotate each occurrence of H-G pairs in abstracts. When a sentence contains more than one hypertension name and more than one gene name, the system makes sufficient copies of the sentence to accommodate all possible H-G pairs. We call these copies H-G pair instances. Each instance is a candidate for a relation between a hypertension entity and a gene mention. After machine annotation is complete, our in-lab biologists verified the results with an inter-annotator agreement of 97.2%. Table 2 shows an example of an annotated sentence.

**Table 2:** Example of annotated sentence

Sentence	KeyRelation?
In conclusion, <GENE> REN 10631A alleles</GENE> are significantly associated with <DISEASE> EHT</DISEASE> in the Emirati population.	Yes

Furthermore, to examine the performance of our proposed approach in a different disease domain, we chose diabetes and again used GAD to retrieve relevant abstracts. In total, we retrieved 519 sentences from 50 randomly selected abstracts, containing 369 diabetes-gene pairs (D-G pairs). All retrieved abstracts' MeSH terms were tagged with "diabetes" in GAD. In addition to the positive dataset, we compiled a negative dataset of abstracts irrelevant to diabetes as follows: First, we searched PubMed using the keywords query "diabetes" and filtered out the genes or abstracts recorded in GAD. Our biologists then carefully read the filtered results and randomly selected 50 which were not related to diabetes. By combining the negative dataset with the first set, we built a corpus with 451 D-G pairs among 1057 sentences.

### Experiment design

We conducted two experiments to evaluate the proposed system. The first experiment is designed to measure the effects of using proposed features on the system's performance. To test if the enhanced system outperforms the baseline system significantly, we compiled 30 unique training and set sets. To compile each set, we randomly selected 90% of the abstracts in our dataset as the training set and then used the remaining 10% as the test set. We summed the scores for these 30 sets and calculated the averages to compare performance. The second experiment examines the performance gap when the model trained in the hypertension domain is used in the domain of diabetes.

Finally, we report some preliminary results of H-G pair extraction on real-world data.

### Evaluation metrics

As for evaluating the hypertension candidate gene extraction system, since it output a ranked list of hits, the AUC of interpolated Precision/Recall (iP/R) curves was chosen as the evaluation scheme. As additionally discussed below, the AUC ***A ***of the interpolated P/R function ***f***_*pr *_is defined as follows:

Where ***n ***is the total number of correct hits and ***p***_*i *_is the highest interpolated precision for the correct hit ***j ***at ***r***_*j*_, the recall at that hit. Interpolated precision ***p***_*i *_is calculated for each recall ***r ***by taking the highest precision at ***r ***or any ***r' > r***.

In other words, for each ***j***^th ^ranked result, the current ***r ***is calculated, and for each distinct ***r ***value, the highest ***p ***(N.B., this is not the ***p***_*i*_) at that ***r ***is retained. This results in a jigsaw like curve from high P at low R to the lowest P of the system at its highest R. The ***p ***at ***r ***then is interpolated if there is a higher ***p ***at any higher ***r***, producing a step-like curve from the highest precision measured at the first correct hit in the ordered result down to the lowest precision at the highest recall when traversing the result list by rank.

## Results

### Experiment 1: the performance of the proposed features

We examine the detailed statistics of all the experiments in Table 3. In the table, in addition to , we also list the sample standard deviation of the AUC score (). We apply a two-sample *t *test to examine whether one configuration is better than the other with statistical significance. The null hypothesis, which states that there is no difference between the two configurations, is given by

**Table 3:** Performance improvement achieved by each feature set

Config	Baseline Features	Template Features	Positional Features			ΔAUC	*t*	AUC>AUC_B_? (*t *>1.67?)
Baseline	+			0.4936	0.1261	N/A	N/A	N/A
B+T	+	+		0.5133	0.1057	0.0114	0.65	No
B+P	+		+	0.8140	0.087	0.3604	11.44	Yes
B+P+T	+	+	+	0.8184	0.084	0.3783	11.75	Yes

where *μ*A is the true mean F-score of configuration A, *μ*_B _is the mean of configuration B, and the alternative hypothesis is

A two-sample *t*-test is applied since we assume that the samples are independent. As the number of samples is large and the standard deviations of the samples are known, the following *t*-statistic is appropriate:

If the resulting *t *score is equal to or less than 1.67 with a degree of freedom of 29 and a statistical significance level of 95%, the null hypothesis is accepted; otherwise it is rejected.

Here, we first compare the retrieval performance achieved by individually adding template features (B+T) and position features (B+P) to the baseline system (B). We then examine their combined performance (B+T+P). In Table 3, we can see that both template and position features improve the baseline's retrieval performance. Using position features can almost double the original AUC score (0.3604), while template features only result in a marginal improvement (0.0114). Including both enhances retrieval performance to 0.8169, indicating that they are complementary and not have overlapping effects.

### Experiment 2: the performance for extracting diabetes-gene pairs

For comparison, we developed two naive relation extraction methods: (1) treat all recognized genes in the Results or Conclusion section as candidates; and (2) if a gene co-occurs with "diabetes" terms in one sentence of the Results or Colclusion section, treat it as a candidate. In all cases, we rank the candidate genes according to their frequencies.

As shown in Table 4, our best configuration of Exp. 1 significantly outperforms the four naive methods. Furthermore, compared to the performance reported in Experiment 1, the performance slightly increases by 1.16% in diabetes domain. The results show that our method is useful and general enough to extract disease-gene relation pairs in different disease domains.

### Experiment 3: the extraction results on real-world data

We have set up a system to process the real-world data from PubMed. The system uses the query term, "hypertension", to retrieve abstracts from PubMed, and then applies our relation extraction technique to extract H-G pairs.

To measure the actual performance on real-world data, our in-lab biologists checked the extracted genes with PubMed IDs from 1602574 to 18504326 (published from 2005, Feb to 2008, Jul), and carefully examined the context surrounding the H-G pairs to determine whether the candidates were hypertension-related genes or not. Based on 100 samples, we achieved a satisfactory AUC score of 0.8319. Table 5 lists the preliminary results examined by our biologists. Note that the genes listed in Table 5 are new candidates extracted by our system which were not recorded in GAD.

**Table 4:** Diabetes-gene pair extraction performance

Config	Precision	Recall	AUC
(1)	0.3652	0.7925	0.77
(2)	0.5679	0.8679	0.6866
B+P+T	0.6522	0.8491	0.8300

**Table 5:** List of examined genes extracted from real-world data

PubMed ID	EntrezGene ID
16380460	2200
16530037	11117
16540569	10371
16685211	7222
16690767	6523
16801480	116985
16915036	3481
17015768	51320
17182005	2952
17250807	3606
17351372	7222
17351372	7224
17921333	27302
17976639	7178
17986358	8490
18067551	50848
18075463	2185
18097620	6098
18156195	5594
18156195	3726
18156195	1385
18158339	2697
18360038	8601
18360038	9630
18398332	7293
18398344	8837

## Discussion

In this section, we discuss possible reasons for how our most effective feature set, section features work. We then analyze key gene distribution over the four sections of an abstract. Finally, we conduct an error analysis.

### Performance gained using section categorizer

**Figure 4:**
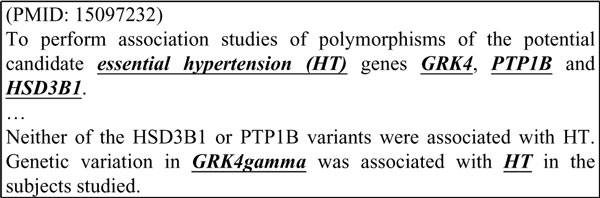
**Section categorization**.

After analyzing our experimental results, we found that the section categorizer could effectively identify an abstract's key H-G pairs. Take Figure 4 for example. The sentence before "......" belongs to the abstract's objective section, while the sentence after "......" is part of its Results section. Originally, all H-G pairs in the abstract were extracted, but after using the section categorizer, only the key H-G pair (HT, GRK4gamma) is retained.

### Analysis of key hypertension genes

**Figure 5:**
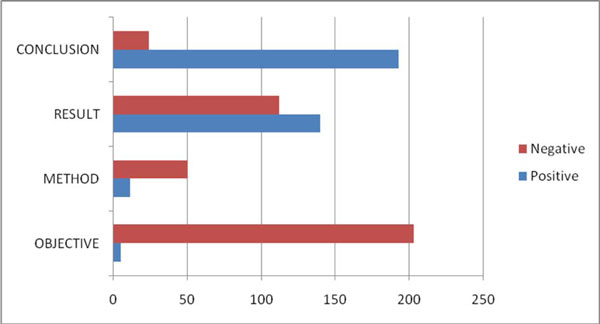
**The distribution of key hypertension genes in different sections**.

We counted all the key hypertension genes appearing in different abstract sections in our GAD corpus. Figure 5 illustrates the distribution. We can see that approximately 95% of all key genes are in the RESULTS or CONCLUSION sections. For example, in the CONCLUSION section, there are a total of 193 key hypertension genes versus only 24 non-key genes. This distribution clearly supports implementation of our proposed section features.

### Error analysis

We summarized the errors into the following main types.

1. Errors caused by incorrectly tagged NEs

NER errors reduce the precision of our system greatly because non-gene entities were incorrectly included in candidate H-G pairs, especially those NE types that are frequently appear in hypertension articles. For example, chemical compound NEs, such as TZDs (thiazide diuretics) and single-nucleotide polymorphism (SNP) NEs, such as "VNTR" and "825T allele" are two main NE types that are incorrectly identified as gene names. We believe constructing NE recognizers that can identify both types may effectively diminish such false positives.

2. Errors caused by incorrectly categorized sections

**Figure 6:**
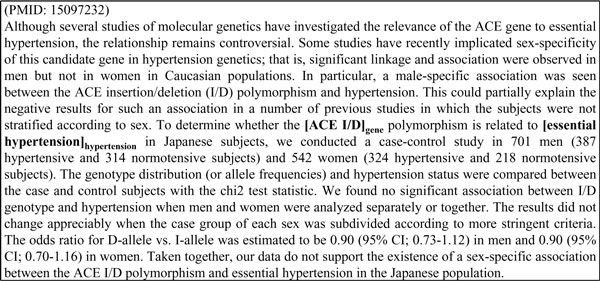
**An incorrectly sectioned abstract**.

The analysis of the distribution of key genes over sections explains why the proposed position features are effective for determining key hypertension genes. Unfortunately, not all abstracts are written following the normal Objective-Methods-Results-Conclusion flow. Sections in such abstracts may be misclassified. Our system may therefore be misled by these incorrect section features. Take the H-G pair (ACE I/D, essential hypertension) in the article shown in Figure 6 for example. We can see that this abstract are not explicitly sectioned. In the feature generation step, our section categorizer classifies *S *as part of the Results section. In fact, *S *should be in the Methods section. Although the uncertain meaning of the surrounding word "whether" indicates that this H-G pair is not a key H-G relation in this article, it is still tagged as a key relation because of the strong influence brought by its section feature. One possible solution is to collect the patterns that describe uncertain relations or relations in previous studies and to exploit such information in key gene extraction.

## Conclusion

In this paper, we have proposed a supervised learning approach for extracting key hypertension-related genes using a maximum entropy model which achieves a satisfactory AUC score of 81.84%. In addition to traditional word, chunk, and parser features, we formulated and tested template and position features. The template features can distinguish true H-G pairs, while position features improve the accuracy of key H-G pair identification. Most importantly, we found that using position features can almost double the original AUC score. Although not nearly as effective as position features, template features still result in a marginal improvement.

Our system is the first to not only extract all genes related to a specific disease in an abstract but also rank them according to their relevance to the key findings of the paper. We achieved this by successfully integrating machine learning models and pattern matching. Moreover, our templates are automatically generated thus significantly reducing manual effort.

To the best of our knowledge, this the first systematic study of extracting hypertension-related genes and the first attempt to create a hypertension-gene relation corpus based on the GAD database. Furthermore, our paper proposes and tests novel features for extracting key hypertension genes, such as relative position, section, and template features, which could also be applied to key-gene extraction for other diseases.

## Competing interests

The authors declare that they have no competing interests.

## Authors' contributions

RTH Tsai designed all the experiments and wrote most of this paper. HJ Dai and WL Hsu discussed and refined the paper. PT Lai wrote the key hypertension-related gene extraction system and conducted all experiments. HJ Dai wrote the section categorization system. CH Huang, YY Bow, and YC Chang, the three biologists in our laboratory, annotated the GAD corpus. RTH Tsai guided the whole project.

## Note

Other papers from the meeting have been published as part of *BMC Genomics *Volume 10 Supplement 3, 2009: Eighth International Conference on Bioinformatics (InCoB2009): Computational Biology, available online at http://www.biomedcentral.com/1471-2164/10?issue=S3.
